# Comparative analysis of single vs. double cage insertion in unilateral biportal endoscopic lumbar interbody fusion: clinical and radiological outcomes

**DOI:** 10.3389/fsurg.2026.1784194

**Published:** 2026-05-25

**Authors:** Guisi Xie, Yanli Pan, Zhongshu Shan, Chan Wang Lei, Lek Hang Cheang, Jiaming Liang, Junfeng Shen, Wei Zhang, Chengyue Zhu

**Affiliations:** 1 Department of Orthopaedics, Hangzhou Lin’an Traditional Chinese Medicine Hospital/ Lin’an Branch of Hangzhou Traditional Chinese Medicine Hospital, Hangzhou, China; 2 Department of Orthopaedics, Hangzhou Traditional Chinese Medicine Hospital, Hangzhou, China; 3 Hangzhou Clinical Medical College, Zhejiang Chinese Medical University, Hangzhou, China; 4 Department of Orthopaedics, Qinghai Provincial People’s Hospital, Xining, China; 5 Department of Orthopaedics, Conde S. Januário Hospital, Macao, Macao SAR, China

**Keywords:** clinical outcome, interbody cage, lumbar degenerative disease, lumbar interbody fusion, unilateral biportal endoscopy

## Abstract

**Objective:**

To compare clinical and radiological outcomes of single vs. double cage insertion in unilateral biportal endoscopic lumbar interbody fusion (UBE-LIF) for lumbar degenerative diseases (LDD).

**Methods:**

This retrospective study analyzed 103 LDD patients undergoing UBE-LIF from June 2021 to June 2023, with 1:3 matching for age, sex, and operative level, forming unilateral cage (Group S, *n* = 51) and bilateral cage (Group D, *n* = 17) groups. Outcomes included fusion rates (assessed by lumbar CT at 1 and 2 years), perioperative metrics (surgical time, estimated blood loss [EBL], hospital stay), postoperative cage subsidence, complications, and patient-reported outcomes (VAS back/leg, ODI, SF-12 PCS). Statistical analyses used Student's t-tests and Fisher's exact tests (*p* < 0.05).

**Results:**

Groups were similar in age, sex, BMI, smoking status, diabetes, and fusion level, except Group D had lower BMD (−1.7 ± 0.3 vs. −1.3 ± 0.5, *p* = 0.005). Group D had longer surgical time (204.1 ± 36.1 vs. 157.6 ± 16.2 min, *p* < 0.001) and higher EBL (150.0 ± 76.8 vs. 106.8 ± 26.8 mL, *p* = 0.001). Postoperative cage subsidence was significantly more frequent in the single-cage group (17/51, 33.3%) than in the double-cage group (1/17, 5.9%; *p* = 0.029). Fusion rates were comparable at 1 year (94.1% vs. 88.2%, *p* = 0.675) and 2 years (100% vs. 98.0%, *p* = 1.000), with Group D showing a numerically higher 1-year rate. Group D had lower VAS back scores at 6 months (1.8 ± 0.9 vs. 2.4 ± 1.0, *p* = 0.041). Complication rates and hospital stay were similar.

**Conclusion:**

Single-cage UBE-LIF offers comparable fusion rates and clinical outcomes with reduced surgical time and blood loss, despite a higher incidence of cage subsidence. Double-cage insertion may provide advantages in early stability and lower subsidence risk, making it preferable in select patients with lower BMD. Both techniques yield favorable long-term results in UBE-LIF for lumbar degenerative diseases.

## Introduction

Lumbar degenerative diseases (LDD), including lumbar disc herniation, lumbar spinal stenosis, and degenerative spondylolisthesis, represent a significant cause of disability, often resulting in chronic low back pain and neurological impairment (1). Surgical intervention, particularly lumbar interbody fusion (LIF), remains a cornerstone in the treatment of LDD, aimed at stabilizing the spine, decompressing neural structures, and restoring disc height (2). Traditional LIF procedures, such as posterior lumbar interbody fusion (PLIF) and minimally invasive transforaminal lumbar interbody fusion (MI-TLIF), have demonstrated efficacy; however, they are often associated with extended recovery times, significant soft tissue disruption, and a higher incidence of complications (3).

In recent years, the introduction of new minimally invasive techniques, including unilateral biportal endoscopic lumbar interbody fusion (UBE-LIF), has revolutionized the approach to lumbar fusion surgery (4). The UBE technique utilizes two independent portals, one for visualization and the other for manipulation, providing enhanced surgical views, reduced tissue disruption, and potentially shorter recovery periods compared to traditional open and percutaneous approaches (5). Despite these advantages, the choice between single or double cage insertion during UBE-LIF remains a subject of debate. While the single cage approach is associated with a minimally invasive profile, double cages are thought to provide greater mechanical stability and a broader fusion surface, which may improve the likelihood of successful fusion (6). Studies have demonstrated comparable clinical outcomes between UBE-LIF and conventional PLIF or MI-TLIF, with some suggesting that UBE-LIF may offer improved recovery metrics, such as reduced blood loss and shorter hospital stays (7–9). However, the impact of cage configuration (single vs. double) on clinical outcomes, including fusion rates, complications, and long-term stability, has not been explored in the literature.

This study aims to compare the clinical and radiological outcomes of single vs. double cage insertion in UBE-LIF for the treatment of LDD. By analyzing patient data, surgical outcomes, and fusion rates, we seek to determine the optimal approach for improving patient outcomes and minimizing complications in minimally invasive lumbar spine surgery. The results of this study will contribute to the growing body of evidence guiding surgical decision-making in UBE-LIF procedures and inform clinical practice regarding cage selection.

## Methods and materials

### Patient population

Informed patient consent and Institutional Review Board approval were obtained. This retrospective study included consecutively enrolled patients who underwent UBE-LIF for LDD between June 2021 and June 2023. A total of 103 patients were initially identified. Due to an unbalanced ratio between double (group D, *n* = 17) and single (group S, *n* = 51) cage insertions, a 1:3 cohort matching was performed based on age, sex, and operative level (Table 1). All patients received bilateral pedicle screw fixation for degenerative spinal pathologies such as lumbar disc herniation with endplate inflammation, spinal stenosis, or degenerative spondylolisthesis (Table 2). Inclusion criteria were as follows: Primary, single-level lumbar interbody fusion performed under UBE guidance. Bilateral pedicle screw instrumentation. Preoperative diagnosis of degenerative degenerative disease. Exclusion criteria included: absence of interbody cage implantation or use of expandable cages. Spinal trauma, infection, neoplastic lesions, or prior lumbar surgery at the index level. All surgeries were performed by the same surgical team at a single academic institution.

**Table 1:** Patient demographics.

Index	Double cage (*n* = 17) *N* (%)	Single cage (*n* = 51) *N* (%)	*p*
Age (years)	65.3 ± 4.7	62.8 ± 5.5	0.104
BMI	24.4 ± 1.9	25.0 ± 2.2	0.363
BMD	−1.7 ± 0.3	−1.3 ± 0.5	0.005*
Surgical time	204.1 ± 36.1	157.6 ± 16.2	0.000*
EBL	150.0 ± 76.8	106.8 ± 26.8	0.001*
Hospital stay	6.8 ± 1.1	6.3 ± 1.1	0.149
Gender			1.000
Male	12 (70.6)	36 (70.6)	
Female	5 (29.4)	15 (29.4)	
Smoking			1.000
Smoker	8 (47.1)	23 (45.1)	
Nonsmoker	9 (52.9)	28 (54.9)	
Diabetes			0.669
Dibetic	1 (5.9)	7 (13.7)	
Nondiabetic	16 (94.1)	44 (86.3)	
Fusion level			0.910
L3/L4	3 (17.6)	8 (15.7)	
L4/L5	10 (58.8)	29 (56.9)	
L5/S1	4 (23.6)	14 (27.4)	
Fusion-1y			0.675
Solid fusion	16 (94.1)	45 (88.2)	
Nonunion	1 (5.9)	6 (11.8)	
Fusion-2y			1.000
Solid fusion	17 (100)	50 (98)	
Nonunion	0 (0)	1 (2)	
Complications(Y/N)			0.669
Dural tear	1 (5.9)	2 (3.9)	
FBSS	0	3 (5.9)	
Infection	0	1 (2)	
Cage subsidence	1 (5.9)	17 (33.3)	0.029

Values are presented as number (%) or mean ± standard deviation. BMI, body mass index. BMD, bone mineral density. EBL, estimated blood loss). FBSS, failed back surgery syndrome.

* *p* < 0.05, statistical significance.

**Table 2:** Etiological distribution.

Diagnosis	Group D (*n* = 17)	Group S (*n* = 51)	Total
Spondylolisthesis	10 (58.8%)	28 (54.9%)	38 (55.8%)
Lumbar spinal stenosis with instability	4 (23.5%)	15 (29.4%)	19 (27.9%)
Disc herniation with Modic type 1 changes	3 (17.6%)	8 (15.7%)	11 (16.2%)
Total	17 (100%)	51 (100%)	68 (100%)

Values are presented as number (%). *p* = 0.878 by Fisher's exact test for overall distribution difference between groups.

### Surgical technique

All procedures were performed under general anesthesia with the patient placed prone on a radiolucent table. Two ipsilateral skin incisions were made at the lateral borders of the pedicles to establish the viewing and working portals. After sequential dilation and insertion of the endoscope and instruments, continuous saline irrigation was applied throughout the procedure.

**Figure 1:**
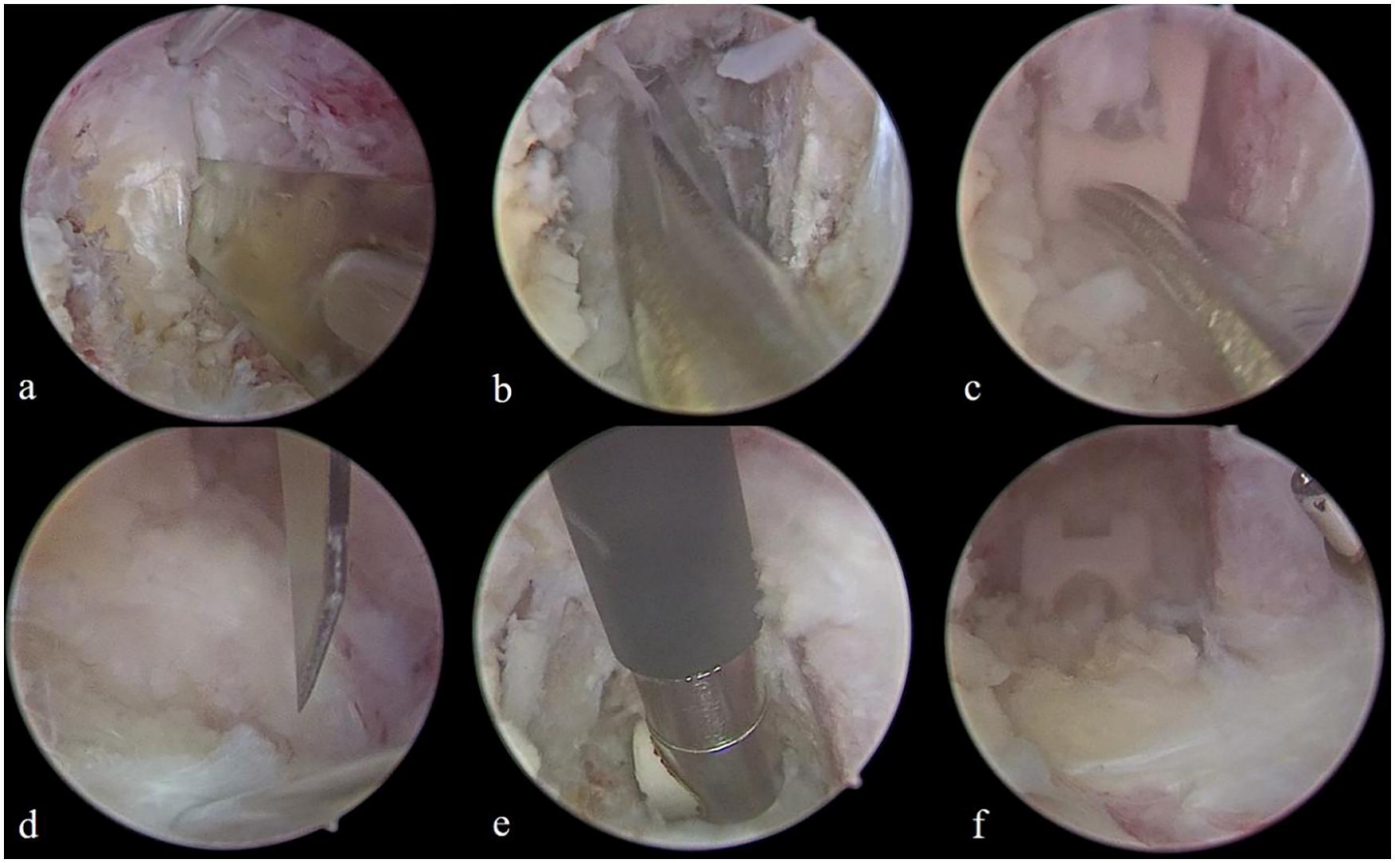
**(a,d)** A 1.5-mm Kirschner wire is used as a nerve retractor to protect the traversing nerve root, with a sharp blade employed to incise the annulus fibrosus. **(b,e)** Endplate preparation under endoscopic visualization on the ipsilateral and contralateral sides. **(c,f)** Longitudinal insertion of a single cage on the ipsilateral and contralateral sides, respectively.

**Figure 2:**
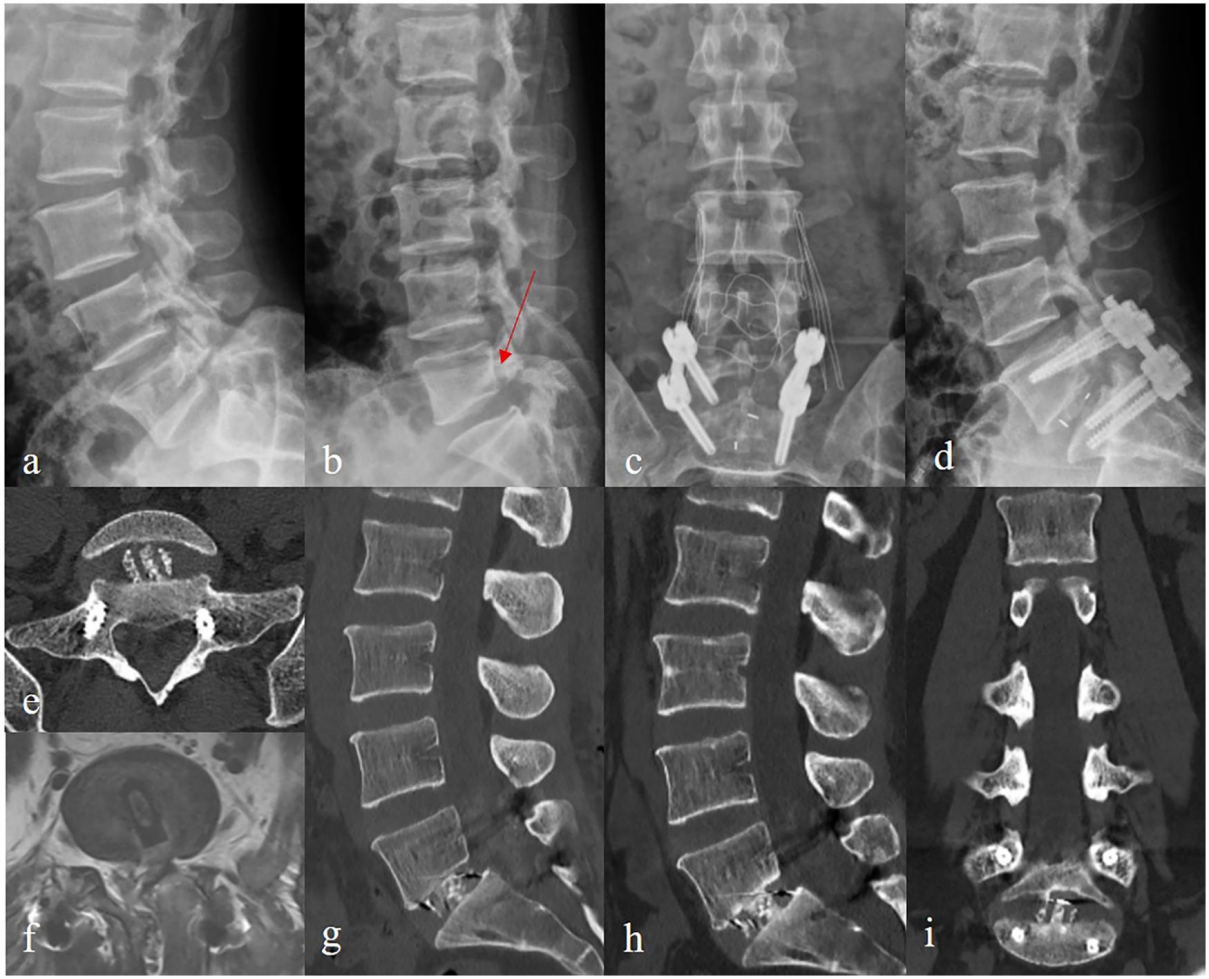
**(a,b)** A 50-year-old male patient showed L5 isthmic spondylolisthesis with dynamic instability on flexion-extension x-rays (translation >3 mm and angulation >10°). **(c–f)** Postoperative imaging (x-ray, CT, and MRI axial views) at 2 days post-UBE-LIF, demonstrating satisfactory internal fixation and one cage in position. **(g)** CT at 6 months post-surgery showing no solid fusion at L5-S1. **(h,i)** CT at 1 year post-surgery confirming solid fusion at L5-S1.

Ipsilateral decompression was carried out via laminotomy and facetectomy using a high-speed burr and Kerrison rongeur. The intervertebral disc was removed, and endplate preparation was performed under direct endoscopic visualization (Figure 1). In the single cage group, an appropriate size cage filled with autologous bone was inserted through the ipsilateral working portal into the disc space under fluoroscopic guidance (Figure 2).

**Figure 3:**
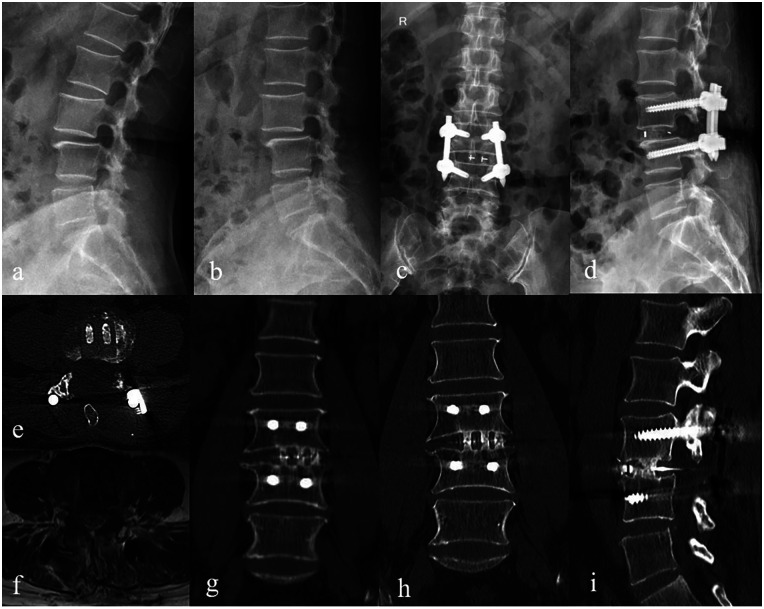
**(a,b)** A 58-year-old female patient with refractory low back pain and intermittent claudication, exhibiting L3 degenerative spondylolisthesis and lumbar instability on x-ray. **(c–f)** Postoperative imaging (x-ray, CT, and MRI axial views) at 2 days post-UBE-LIF, demonstrating satisfactory internal fixation and two cages in position. **(g)** CT at 3 months post-surgery showing no solid fusion at L3-L4. **(h,i)** CT at 6 months post-surgery confirming solid fusion at L3-L4.

In the double cage group, after completing ipsilateral decompression and discectomy, a third auxiliary portal was created over the base of the contralateral superior articular process (“Zhang's portal”) under endoscopic guidance (10). Contralateral flavectomy, laminotomy, and endplate preparation were performed through this portal. A retractor was inserted via the ipsilateral working portal to protect the contralateral traversing nerve root. A second cage was then inserted through the contralateral portal into the prepared disc space (Figure 3). Finally, percutaneous bilateral pedicle screw fixation was completed, and drainage tubes were placed before closure.

### Data collection

Demographic and perioperative variables were collected, including age, sex, body mass index (BMI), bone mineral density (BMD, T-score), smoking status and presence of diabetes.

**Figure 4:**
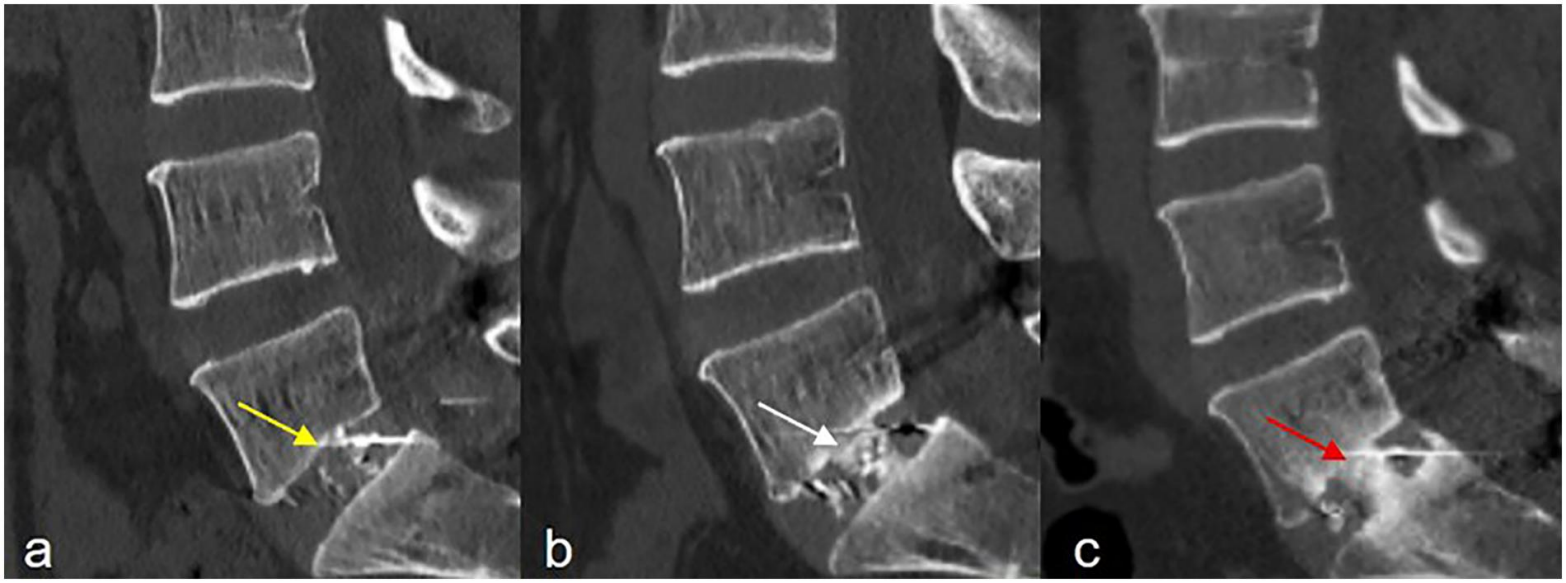
Grading definitions of the mBSF scale. **(a)** Grade I (Radiographic Pseudarthrosis): Presence of definitive signs of pseudarthrosis, such as a radiolucent zone surrounding the cage and the absence of bone bridge formation (Yellow arrow). **(b)** Grade II (Uncertain Fusion): Atypical radiographic findings where fusion status cannot be clearly determined; may be accompanied by partial callus formation or blurred borders (White arrow). **(c)** Grade III (Radiographically Stable Fusion): Demonstration of continuous trabecular bone bridging across the fusion space, with no radiolucent lines between the cage and endplates, indicating that solid bony fusion has been achieved (Red arrow).

**Figure 5:**
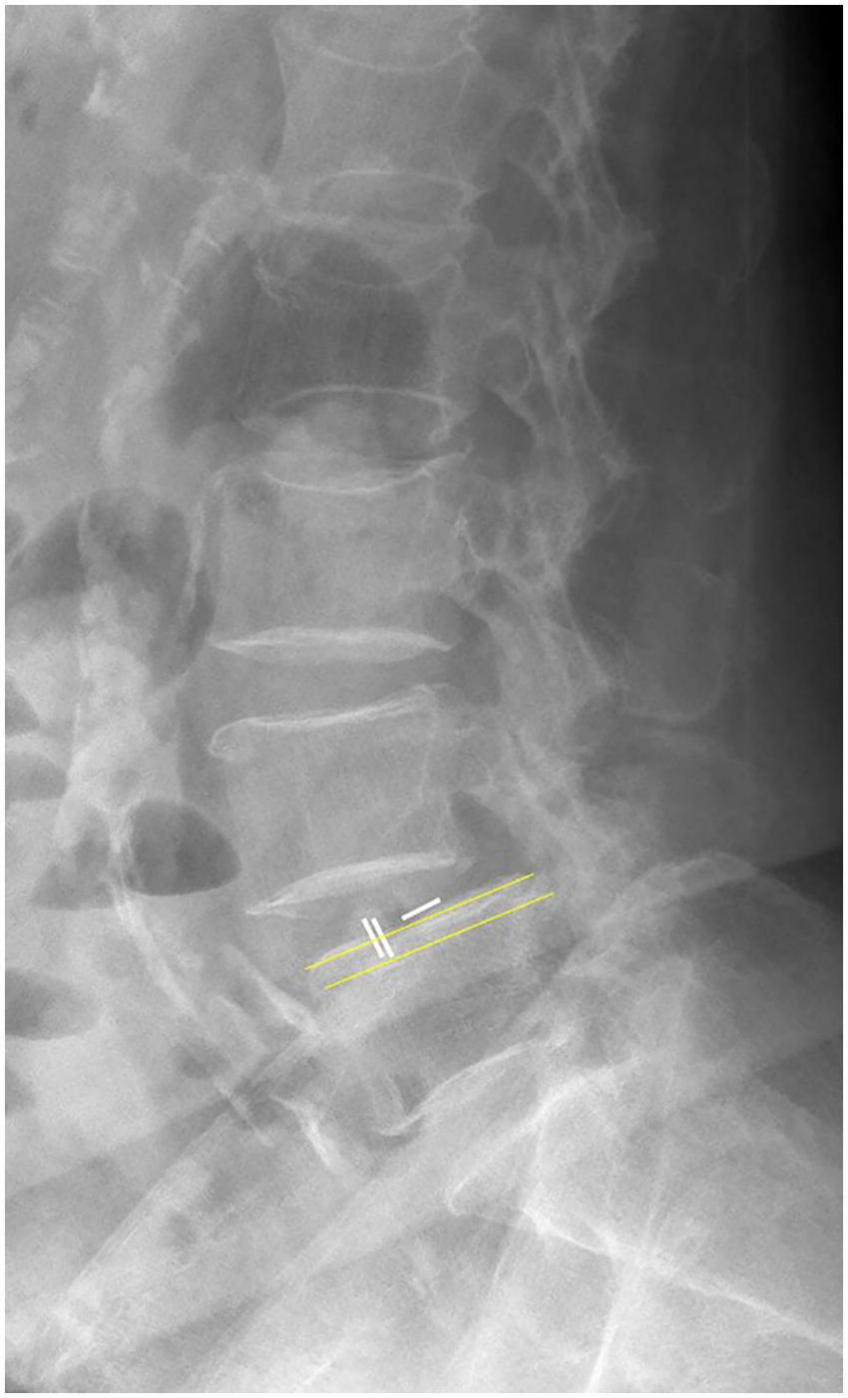
A horizontal reference line is drawn tangential to the superior vertebral endplate, and a second parallel line is drawn at the level of the cage markers that have migrated into the endplate; a vertical distance exceeding 2 mm between these two parallel lines is defined as cage subsidence.

Perioperative parameters included operative time (minutes), estimated blood loss (mL), and length of hospital stay (days), fusion state, cage subsidence and complications. Estimated blood loss (EBL) was calculated by the attending anesthesiologist and surgical team as follows: total volume in the suction canister minus the volume of irrigation fluid used, plus the weight difference of soaked sponges/gauzes (assuming 1 g ≈ 1 mL blood) (11, 12). Due to continuous saline irrigation in UBE-LIF, we carefully collected all effluent and subtracted irrigation volume to minimize overestimation. Fusion status was assessed on thin-slice lumbar CT scans at 1- and 2-year follow-up using the modified Brantigan-Steffee-Fraser (BSF) scale (13). Solid fusion (Grade III) was defined as continuous trabecular bridging bone across >50% of the disc space without radiolucent lines around the cage or implant migration (Figure 4). Cage subsidence was assessed on postoperative lateral view on x-ray and defined as ≥2 mm migration of the cage into the superior or inferior vertebral endplate (14) (Figure 5).

Patient-reported outcome measures were collected preoperatively and at 6 weeks, 12 weeks, 6 months, 1 year, and 2 years postoperatively. These included the visual analog scale (VAS) for back and leg pain, the Oswestry Disability Index (ODI), the 12-item Short Form Health Survey physical component summary (SF-12 PCS). For outcomes with significant baseline differences (e.g., BMD, pre-op ODI), analysis of covariance (ANCOVA) was performed with the baseline value as covariate. Multivariate regression was not performed due to the small sample size in Group D, but we discussed the potential impact of lower BMD in Group D in the Discussion.

4. All statistical analyses were performed using SPSS software (version 21.0; IBM Corp., Armonk, NY, USA). Patients were stratified into two groups according to the type of cage placement (single vs. double). Continuous variables were compared using Student's t-tests, and categorical variables were analyzed using chi-square or Fisher's exact tests. A *p*-value < 0.05 was considered statistically significant.

## Results

Patient demographics, including age (Group B: 65.3 ± 4.7 years vs. Group S: 62.8 ± 5.5 years, *p* = 0.104), BMI (24.4 ± 1.9 vs. 25.0 ± 2.2, *p* = 0.363), gender (M/F: 12/5 vs. 36/15, *p* = 1.000), smoking status (Y/N: 8/9 vs. 23/28, *p* = 1.000), diabetes (Y/N: 1/16 vs. 7/44, *p* = 0.669), and fusion level (L3/L4, L4/L5, L5/S1: 3/10/4 vs. 8/29/14, *p* = 0.910) showed no significant differences, except for bone mineral density (BMD, −1.7 ± 0.3 vs. −1.3 ± 0.5, *p* = 0.005). Perioperative outcomes indicated significantly longer surgical time (204.1 ± 36.1 vs. 157.6 ± 16.2 min, *p* < 0.001) and higher estimated blood loss (EBL, 150.0 ± 76.8 vs. 106.8 ± 26.8 mL, *p* = 0.001) in Group D compared to Group S, with no difference in hospital stay (6.8 ± 1.1 vs. 6.3 ± 1.1 days, *p* = 0.149). Postoperative cage subsidence, defined as ≥2 mm migration of the cage into the superior or inferior vertebral endplate on CT, was significantly more frequent in the single-cage group (17/51, 33.3%) than in the double-cage group (1/17, 5.9%; *p* = 0.029). Solid fusion rates at 1 year (94.1% vs. 88.2%, *p* = 0.675) and 2 years (100% vs. 98.0%, *p* = 1.000) were statistically comparable, though Group D exhibited a numerically higher fusion rate at 1 year (Table 1). Complication rates were similar (6.3% vs. 11.8%, *p* = 0.669). Patient-reported outcomes (VAS back, VAS leg, ODI, SF-12 PCS) improved significantly post-operation in both groups. Notably, Group D had a significantly lower VAS back score at 6 months (1.8 ± 0.9 vs. 2.4 ± 1.0, *p* = 0.041), while Group S had a higher preoperative ODI (39.7 ± 6.6 vs. 35.7 ± 6.9, *p* = 0.035), with no significant differences at other time points (Table 3).

**Table 3:** Patient-reported outcomes.

Index	Doublel cage (*n* = 17)	Single cage (*n* = 51)	*p*
VAS back			
Pre-op	5.2 ± 1.1	4.7 ± 1.0	0.134
6w-postoperation	2.7 ± 1.0	2.6 ± 1.0	0.670
12w-postoperation	3.6 ± 1.2	3.5 ± 1.0	0.732
6m-postoperation	1.7 ± 0.9	2.5 ± 1.0	0.028*^†^
1y-postoperation	1.4 ± 0.9	1.6 ± 1.1	0.506
2y-postoperation	1.5 ± 1.1	1.6 ± 1.0	0.736
VAS leg			
Pre-op	5.8 ± 1.1	5.6 ± 1.1	0.520
6w-postoperation	1.8 ± 0.7	2.2 ± 1.0	0.150
12w-postoperation	2.2 ± 1.1	1.9 ± 1.2	0.482
6m-postoperation	1.4 ± 1.1	1.5 ± 1.0	0.622
1y-postoperation	1.2 ± 1.0	1.3 ± 1.0	0.571
2y-postoperation	1.0 ± 0.6	1.0 ± 0.9	0.867
ODI			
Pre-op	35.7 ± 6.9	39.7 ± 6.6	0.035*
6w-postoperation	26.9 ± 3.9	28.3 ± 5.0	0.282
12w-postoperation	20.6 ± 3.6	21.4 ± 4.1	0.466
6m-postoperation	22.2 ± 4.0	23.3 ± 5.1	0.396
1y-postoperation	17.4 ± 3.8	15.8 ± 4.0	0.145
2y-postoperation	12.2 ± 6.5	13.2 ± 6.0	0.541
SF-12 PCS			
Pre-op	34.5 ± 6.8	33.3 ± 5.9	0.463
6w-postoperation	13.8 ± 4.6	12.6 ± 5.2	0.378
12w-postoperation	9.0 ± 4.7	11.5 ± 5.2	0.088
6m-postoperation	8.4 ± 4.3	8.7 ± 4.0	0.796
1y-postoperation	8.8 ± 5.1	8.9 ± 4.3	0.950
2y-postoperation	8.3 ± 5.8	6.9 ± 4.1	0.267

Values are presented as mean ± standard deviation. VAS, visual analogue scale; ODI, Oswestry Disability Index; SF-12 PCS, 12-item Short Form health survey physical composite score.

* *p* < 0.05, statistical significance.

† Adjusted *p*-value by ANCOVA using preoperative score as covariate.

## Discussion

The present study aimed to compare the clinical and radiological outcomes of single vs. double cage insertion in UBE-LIF for treating lumbar degenerative diseases. Our findings indicate that while both techniques provided significant improvements in patient-reported outcomes, the double cage approach demonstrated numerically superior early back pain relief (lower VAS back at 6 months), which may suggest better initial segmental stability. However, there were no statistically significant differences in fusion rates, suggesting that both methods are comparable in achieving long-term fusion success. Additionally, the single cage approach exhibited advantages in terms of surgical time and blood loss, making it a more efficient choice in certain patient populations.

The double cage configuration, which showed a numerically higher fusion rate at 1 year (94.1% vs. 88.2%), likely benefited from increased mechanical stability due to the broader interbody surface provided by the cages. This result aligns with existing literature indicating that double cage placement is particularly advantageous for patients with lower bone mineral density (BMD) or those at risk of non-union (15). However, the lack of statistical significance at the 2-year follow-up (100% vs. 98%) suggests that both techniques are ultimately capable of achieving comparable long-term fusion outcomes, provided that endplate preparation and bone grafting are optimized. Importantly, a key advantage of UBE-LIF lies in its meticulous endplate preparation, facilitated by continuous saline irrigation to control bleeding and direct endoscopic visualization, which ensures thorough exposure of the bony endplate without causing damage (16–19). This contrasts with MI-LIF and traditional open LIF, where such precision and minimal trauma to the endplate is often unachievable, potentially contributing to the comparable long-term outcomes observed in both groups.

In the present study, postoperative cage subsidence was significantly more common in the single-cage group (33.3% vs. 5.9%, *p* = 0.029). This finding aligns with previous literature suggesting that unilateral cage placement may concentrate mechanical stress on the endplate, particularly in patients with lower BMD (15). Nevertheless, despite the higher subsidence rate in Group S, long-term fusion rates remained comparable between groups. This favorable outcome is likely attributable to the superior endoscopic visualization and meticulous endplate preparation under continuous saline irrigation in UBE-LIF, which enables precise cage positioning and optimal load distribution even in technically challenging cases. These results further support the clinical equivalence of single- vs. double-cage insertion in UBE-LIF while emphasizing the importance of individualized cage selection when subsidence risk factors are present.

The reduced back pain scores observed at 6 months in the double cage group may reflect the improved segmental stability afforded by the bilateral cages. This is consistent with findings from previous studies on double cage placement in LIF, where enhanced stability has been associated with better early pain relief (20). However, the transient nature of this advantage, with back pain scores converging by 1 year, suggests that other factors, such as patient rehabilitation and long-term spinal mechanics, may play a more significant role in achieving sustained pain relief.

In contrast, the single cage approach demonstrated shorter surgical times (157.6 vs. 204.1 min) and less blood loss (106.8 vs. 150.0 mL), which may be attributed to the simpler surgical procedure that avoids additional portal creation and dissection. These advantages are in line with the goals of minimally invasive spine surgery, which seeks to reduce operative trauma, expedite recovery, and minimize complications (21, 22). However, the potential trade-offs in terms of early mechanical stability and back pain relief emphasize the need for careful patient selection when choosing the surgical approach.

Our results are consistent with several recent studies comparing different cage configurations in LIF. For example, Lee (23) found that both single and double cage configurations provided comparable fusion rates and complication profiles, although the latter offered superior mechanical stability in the early postoperative period. Similarly, studies by Fogel (6) have reported that while double cages may reduce the risk of subsidence and improve stability, single cage placement remains a viable option with fewer complications and faster recovery times.

Furthermore, the use of UBE-LIF in comparison to traditional open fusion approaches has been supported by multiple studies highlighting its benefits, including reduced blood loss, shorter hospital stays, and comparable clinical outcomes. However, as emphasized by Pao (24), the insertion of two cages via a minimally invasive technique, such as UBE-TLIF, offers enhanced outcomes in terms of segmental stability and fusion rates. Our study expands upon this by demonstrating that even in the context of UBE-LIF, the choice of cage configuration can significantly influence both early and long-term clinical outcomes.

For patients with lumbar degenerative diseases, UBE-LIF, whether using a single or double cage configuration, offers a minimally invasive alternative to traditional open fusion techniques. The double cage approach may be preferred for patients with higher mechanical demands, such as those with lower BMD or severe degeneration. Conversely, the single cage technique may be more appropriate for patients requiring quicker recovery, particularly when surgical efficiency is prioritized.

From a theoretical perspective, this study adds to the growing body of evidence supporting the use of UBE-LIF in LDD. By comparing the effects of different cage configurations, we highlight the trade-offs between mechanical stability and surgical efficiency, which should inform clinical decision-making. Additionally, our study reinforces the notion that meticulous surgical technique and patient-specific factors are key determinants of fusion success, regardless of the cage configuration.

Several limitations must be acknowledged. First, despite 1:3 propensity matching on age, sex, and level, the inherent imbalance reflects real-world adoption rates of the more technically demanding double-cage technique. Future studies with larger cohorts and multi-center data would help validate these results and provide more robust evidence regarding the comparative effectiveness of single vs. double cage insertion in UBE-LIF. Additionally, while our study provides insights into short- and medium-term outcomes, longer follow-up is needed to assess the durability of the observed benefits, particularly in terms of fusion rates and back pain relief.

Moreover, while we controlled for several key demographic and surgical factors, other variables, such as bone quality and postoperative rehabilitation, could have influenced the results. Future research should focus on investigating the role of these factors in shaping long-term outcomes, as well as exploring the impact of different cage designs and materials on fusion success and complications.

## Conclusion

Both single and double cage insertion techniques in UBE-LIF yield favorable fusion rates and significant clinical improvements in patients with lumbar degenerative diseases. The double cage approach offers enhanced mechanical stability, particularly in the short term, while the single cage approach provides advantages in terms of surgical efficiency and faster recovery. These findings underscore the importance of individualized treatment plans that consider both mechanical stability and operative factors. Future research with larger, prospective cohorts is needed to further elucidate the long-term benefits and risks associated with each technique.

## Data Availability

The original contributions presented in the study are included in the article/Supplementary Material, further inquiries can be directed to the corresponding author/s.
